# Short Term Outcome of Anterior Lamellar Reposition in Treating Trachomatous Trichiasis

**DOI:** 10.1155/2015/568363

**Published:** 2015-03-31

**Authors:** Rania A. Ahmed, Sameh H. Abdelbaky

**Affiliations:** Ophthalmology Department, Kasr Al Aini Medical School, Cairo University, Cairo 12615, Egypt

## Abstract

*Purpose*. To evaluate the outcome of anterior lamellar reposition (ALR) in treating trachomatous trichiasis. *Methods*. Patients with trachomatous trichiasis or entropion with short tarsus were treated by ALR between February 2009 and November 2013. This included splitting of the lid margin behind the aberrant lash line to separate the lid lamellae. The anterior lamella was recessed and fixated using 4/0 silk sutures. The extra lashes and their routes were excised. Sutures were removed by the 3rd week and patients completed 6 months of follow-up. Recurrence of ≤5 lashes was treated by electrolysis. *Results*. The study included 752 eyelids (445 patients; 58.4% females, 41.6% males), mean age 53.2 ± 6.9 y. 179 (25.1%) lids had entropion while 287 (64.5%) patients had corneal affection. By the third week, 2.66% lid had trichiasis while 30.8% had no rubbing lashes. By the 6th month, 14.9% of lids showed recurrence while 66.1% were completely cured (CI = 0.63–0.69) and 19% had partial success (CI = 0.16–0.21). Abnormal lid appearance persisted in 2.66% and 12.9% required another surgery. *Conclusion*. ALR is a good option for treating trachomatous trichiasis especially without cicatricial entropion. Excision of dysplastic lashes is thought to augment the surgical outcome.

## 1. Introduction

Trachoma is a chronic keratoconjunctivitis caused by an obligate intracellular organism,* Chlamydia trachomatis*. It is the worldwide leading yet avoidable infectious cause of ocular morbidity. The World Health Organization (WHO) estimates that 21.4 million people suffer from active trachoma of which 7.2 million have blinding trichiasis while 1.2 million people are actually blind [1, 2]. The disease is still endemic in Egypt, among 53 countries, as reported by the WHO [3].

This disease is characterized by recurrent attacks of chronic follicular conjunctivitis, progressive conjunctival scarring with subsequent misdirected lashes that, on rubbing against the cornea, is called trichiasis. Trachomatous trichiasis (TT) could also be secondary to metaplastic lashes or cicatricial entropion [4]. In addition to being a source of chronic irritation, TT usually causes a threat to the cornea in the form of recurrent ulceration, corneal opacities, and secondary infection that my progress to corneal melting, perforation with loss of the globe [5].

In 1997, the Alliance for the Global Elimination of Blinding Trachoma by 2020 (GET 2020) was founded. A year later, SAFE strategy called for trachoma elimination. It is the strategy of surgical treatment, antibiotic therapy for acute infection, face cleanliness, and environmental changes to improve sanitation [3].

Nearly all cases of trichiasis need surgical intervention. Conservative observation is appropriate only for few patients who have trichiasis in the far ends of the eyelid where the lashes are not threatening the cornea [6]. Bilamellar tarsal rotation (BLTR) and posterior lamellar tarsal rotation (PLTR) were the recommended procedures by the WHO [3]. However, they were not recommended for treating distichiatic or metaplastic lashes as well as cicatricial entropion cases with defective lid closure due to tarsus shortening, irregular lid margin as well as lids with previous entropion surgery [7].

Anterior lamellar reposition (ALR) with or without mucous membrane grafting was first described by Welsh et al. to correct cicatricial entropion associated with distichiatic cilia yet its use expanded to treating trichiasis and cases of entropion with short or thin tarsus [7, 8]. Other surgical options include direct excision of the lash bearing area and anterior lamella nearby, grey line splitting, and direct follicle ablation in addition to the less invasive options as electrolysis, epilation, cryotherapy, and laser ablation [6].

Although ALR is an established treatment modality, reports about its use in TT especially in cases that are not secondary to cicatricial entropion are far less than reports of the other two commonly used procedures. In this work, the outcome of ALR in TT was evaluated.

## 2. Materials and Methods

This is a prospective noncomparative study that took place from February 2009 to November 2013. Patients suffering from trachomatous trichiasis or entropion with short or thin tarsus were recruited from Kasr Al Aini and El Nour hospital clinics. All affected lids were examined by the slit lamp without distraction to evaluate the lid margin and lash position. Conjunctiva and cornea were thoroughly examined for signs of trachomatous affection and fluorescein test was done.

Patients with 5 or less rubbing lashes or entropion with firm, thick, and long tarsus as well as patients who underwent previous anterior lamellar reposition were excluded. We also excluded patients with nontrachomatous conjunctival scarring, for example, ocular cicatricial pemphigoid or chemical injuries.

All cases were requested to stop systemic antiplatelets or anticoagulants, after their physician consultation whenever applicable, prior to surgery. Patients who had recently epilated the rubbing lashes were deferred till lashes regrew (at least two weeks). A written informed consent was obtained from all patients. The study and data collection conformed to all local laws and complied with the principles of the Declaration of Helsinki.

### 2.1. Surgical Procedure

Topical anaesthesia was installed in both eyes. The addressed lid was then infiltrated in the submuscular space by mixture of Lidocaine HCL 2% and epinephrine 1 : 200.000, and only lidocaine HCL 2% in cardiac or hypertensive patients. The operated side was then prepped and draped.

**Figure 1:**
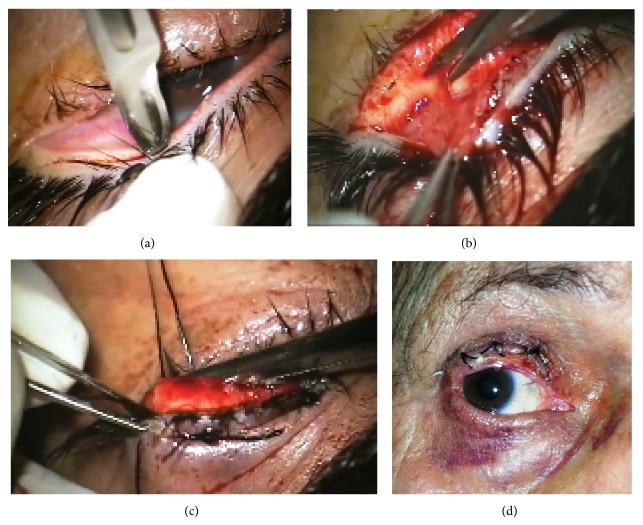
Anterior lamellar reposition in the upper lid: (a) Making the incision behind the abnormal lash line. (b) Separation of the anterior lamella. (c) The anterior lamella is recessed and fixated by 3 sutures. (d) 2 wks. after operation; the granulation tissue is formed, covering the posterior lamella.

Under the surgical microscope, a scratch incision was done along the entire lid margin just behind the abnormal lashes using number 15 bard Parker scalpel blade (Figure 1(a)). The incision was then deepened using the scalpel to create a dissection plane. Undervision dissection was facilitated by asking the assistant to hold the posterior lamella and exert mild traction on the lid while keeping hemostasis. Dissection was then continued in the created plane by Westcott scissors to reach the proper submuscular space in order to separate the anterior and posterior lamellae as far as the peripheral tarsal edge (Figure 1(b)).

In cases of dysplastic lashes that replaced the meibomian orifices in the lid margin, the initial incision had to go through the tarsus. Dissection was then carried out till the routes were exposed then the plane was changed to separate the two lamellae as other cases.

The anterior lamella was recessed (Figure 1(c)) as far as possible and three 4/0 silk transverse mattress sutures were taken. Each suture passed through the lash line to the highest possible point above the tarsus and through the conjunctiva back to the lash line of the anterior lamella to be tied in square knot. The extra-lash bearing skin at the lid margins as well as visible routes embedded in the tarsus was excised. The bare area of the posterior lamella was left to granulate (Figure 1(d)). If both upper and lower lids of the same side were affected, they were operated upon in separate sessions to avoid the possibility of induced ankyloblepharon.

All patients received Tobramycin/Dexamethasone ointment on the lid and intraocular lubricating gel twice per day for ten days. Sutures were removed by the third week. Patients completed at least 6 months of follow-up and were evaluated under the slit lamp by 1 week, 3 weeks, 3 months, and 6 months for lid margin position and abnormalities like notching, necrosis, madarosis, or thickening as well as the condition of conjunctiva and cornea. Recurrence was evaluated as regards the site and the number of the recurrent lashes. For symptomatic patients, rubbing lashes ≤5 detected during the follow-up were removed by electrolysis.

Success was considered when no further surgical intervention was needed. By 6 months postoperatively,* complete* success was defined as no rubbing lashes were detected, even if electrolysis was required once or twice during the follow-up period.* Partial* success was defined in 5 lashes or less that did not require further surgical intervention yet needed more than two sessions of electrolysis or laser ablation.* Failure* was considered when recurrent rubbing lashes were 6 or more and required another surgical intervention. Maldirected, nonrubbing lashes were not considered as recurrence.

Data was collected and analyzed where descriptive statistics were calculated and the numerical data were summarized as mean and standard deviation (±SD), while categorical data were summarized in tables and percentages (%).

## 3. Results

**Figure 2:**
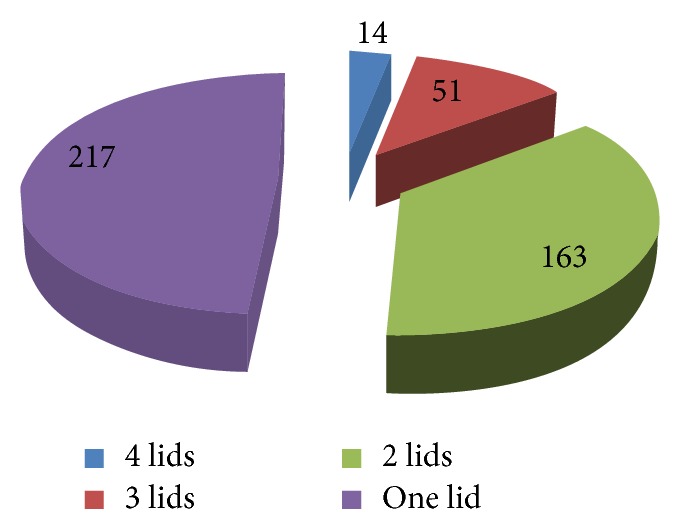
Number of affected lids per patient.

This study included 752 eye lids of 445 patients (58.4% females, 41.6% males) with mean age 53.2 ± 6.9 years. All of the included patients reported repeated epilation of lashes while 163 (36.7%) patients reported previous electrolysis. Associated entropion with thin tarsus was detected in 179 lids (25.1%) and 48.5% of lids had underwent previous surgery (BLTR). All patients had signs of trachoma in the form of pannus siccus, subconjunctival fibrosis, and/or PTDs. Corneal affection was found in 287 patients (64.5%) in the form of nebula or leucoma either localized or diffuse; 89% of these patients (56.8% of the total sample) had bilateral corneal affection. Most of the included patients (48.8%) had only one lid affected. The distribution of number of affected lids per included patients is demonstrated in Figure 2.

**Figure 3:**
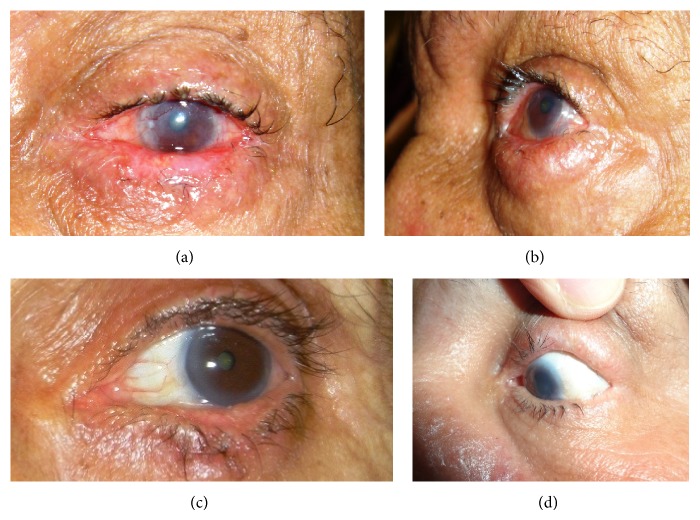
Postoperative outcomes. (a) Thickened lower lid margin 3 wk. postoperatively. (b) The lower lid of the same patient showing return of the anterior lamella to its place, no rubbing lashes with soft appearance of the lid margin. (c) Persistent recession of the anterior lamella. (d) Upper lid temporal madarosis following ALR.

All patients had postoperative lid edema and/or ecchymosis yet all had good lid closure. All lids showed marginal thickening by the 3rd week (Figure 3(a)) that softened over time and disappeared by the 3rd month (Figure 3(b)). The lid had an abnormal appearance in all patients that was present till the time of suture removal by the 2nd week. The lash line migrated back to normal position by the 4th week; however, this abnormal lid appearance persisted in 2.66% of lids (Figure 3(c)).

None of the operated lids showed marginal ischemia or additional corneal lesions in the follow-up period yet localized madarosis developed in 5.72% (Figure 3(d)). Complete success was recorded in 66.09% (95% confidence interval CI = 0.62–0.69) while partial success was recorded in 19.01% (95% CI = 0.16−0.21) making the overall success 85.1% (CI = 0.83–0.88). An overall success rate of 83.8% was also obtained in cases which had previous lid surgery.

**Figure 4:**
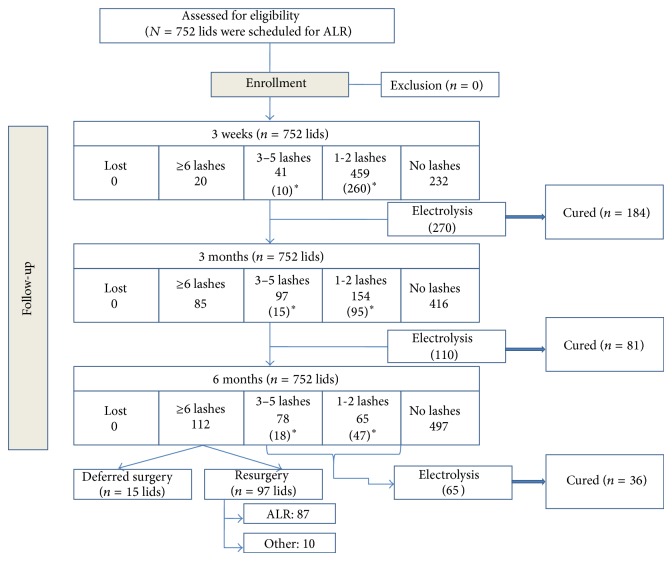
A flow chart illustrating the enrolled lids for intervention and the outcomes per each visit.  ^∗^The number of lids that were exposed to electrolysis.

**Figure 5:**
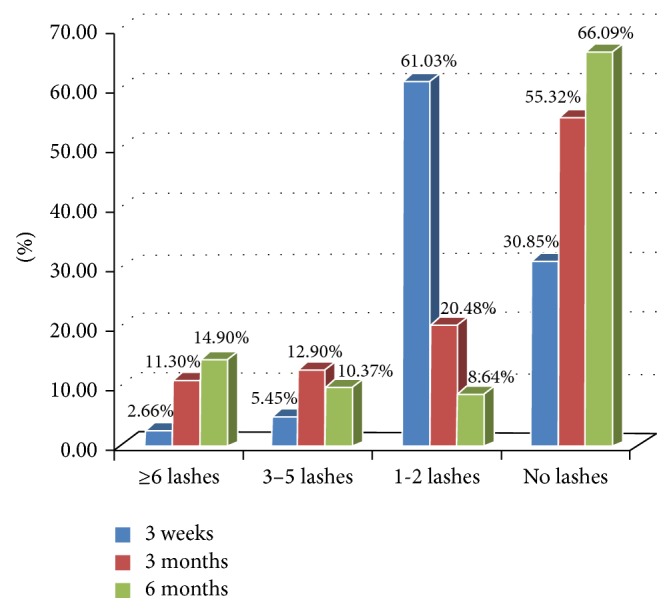
The outcome at each follow-up.

The flow chart in Figure 4 summarizes the outcome numbers throughout the follow-up period as well as any required interventions while the percentages are further illustrated in Figure 5.

By the 3rd week, 270 eyelids required electrolysis with 68.1% cure rate. These recurrent lashes reappeared at their preoperative locations. However, by the 3rd month 110 lids required electrolysis of which 80 lids received their first electrolysis during the follow-up period with 68.8% total cure. Reappearance of lashes in preoperative sites was found in 45% of lids yet the remaining 55% had lashes that appeared in previously lash-free areas. The numbers of lids that received electrolysis along the follow-up period as well as the cure rates are shown in Table 1.

By the end of the follow-up period, 143 lids (19.01% of the whole sample) had recurrence of ≤5 lashes and were considered partial success as none of these lids was enrolled for further surgeries. Sixty-four lids (45.5%) of these recurrences received electrolysis while the rest of cases had the recurrence in the far ends of the lid with no corneal threatening and patients preferred to frequently epilate the recurrent lashes. Recurrence of ≥6 lashes was reported in 112 lids (14.9%) (CI: 0.13–0.17) and was considered to be failure being in need for another surgery. ALR was repeated in 87 lids (77.7% of 112 lids) while 10 lids (8.9%) required another surgical procedure mainly grey line splitting with excision of lash bearing area. Ten patients (15 lids, i.e., 13.4% of failed cases) deferred surgical intervention.

## 4. Discussion

Trichiasis is a painful irritating disease that is usually secondary to conjunctival cicatrizing disorder with subsequent threatening to both vision and globe integrity [5]. Trachoma is still the main cause of trichiasis in many underdeveloped countries [3].

Surgical treatment of TT is a key component of the SAFE strategy supported by the WHO for combating trachoma with direct relation to reducing blindness [9]. BLTR and PTLR are the recommended procedures for treating cases associated with cicatricial entropion via horizontal tarsotomy and everting sutures to rotate the distal end of the lid [10] yet this concept is not effective in cases of trichiasis or distichiasis without entropion [7].

Grey line splitting with anterior lamellar repositioning is an established procedure for treating cicatricial entropion with reported success rate between 75 and 97% [11, 12]. According to the preset definitions in the current study, complete success was achieved in 66.09% of the operated cases while the overall success rate by the end of the follow-up period was 85.1% including complete and partial success. This reflected that further surgical interventions were not necessary.

Similar success rates were achieved by previous studies. Yeung et al. [11] reported the anatomic success rate of 62.5% and functional rate of 75% in their series of 24 lids (CI: 0.44–0.82). Elder and Collin reported anatomical success of 71% in their series of ALR in 16 lids (CI: 0.49–0.93) with ocular cicatricial pemphigoid (OCP) and complete success of 61% [13]. Koreen et al. reported 77% success rate for primary repair in their sample of 35 eyelids (CI: 0.63–0.91); however, their sample included various causes of conjunctival cicatrization [14]. Sodhi et al. reported success rate of 88.4% in their study of 84 eyelids (CI: 0.82–0.95) [8].

On the other hand, some studies reported higher success rates. Kemp and Collin reported an overall success rate of 90.7% in their 183 lids series (CI: 0.88–0.95) [15]. A similar success rate was also reported by Choi et al. [16] in their series that included 30 lower lids with cicatricial entropion. The rate rose to 97% in Hintschich's study of 34 eyelids (CI: 0.91–1.03) [12].

The large sample included in this series compared to the published studies, the additional excision of extra-lash bearing area, the margin of defining success, and the different follow-up periods could explain these different outcomes. The intersurgeon variability was not considered in the current study as both authors received similar surgical training and they adopted the same surgical technique. However, intersurgeon variability was believed to be an important factor for the outcome by both Emerson et al. [17] and Rajak et al. [10]. Hence, it could be presumed to be a contributing factor in explaining the different outcomes in the current study compared to other studies.

Additionally, most studies were concerned about cicatricial entropion in comparison to the current work where cicatricial entropion constituted less than quarter of the included cases. Trachoma is also a unique disease and many of the above mentioned studies included cases of cicatricial entropion due to other causes.

High success rate (83.8%) was reported in cases with history of previous BLTR. Similar results were reported by Sodhi et al. [7] who reported a success rate of 97% of the lids (SS: 66) as a secondary procedure after failed tarsus rotation. The underlying etiology of cicatricial entropion was found to be the major risk factor that significantly influences the surgical outcome of ALR and higher failure rates were associated with infective causes [8]. The severity of the preoperative trichiasis was also found to be a major risk factor for recurrence contrary to presence of preoperative entropion that was considered an independent risk factor [18].

Rajak et al. studied the 4-year overall recurrence of trichiasis and reported 41% recurrence; Three/4 of which occurred in the first 6 months. They referred its causes to disease severity, surgical factors, and the wound healing course [10]. In the current study, 69.14% had residual rubbing lashes by the 3rd week of follow-up mostly 1-2 lashes (61.03%).

Although using the surgical microscope is of utmost importance in this procedure for better visualization, early recurring lashes were actually missed in the primary surgery either because they were fine and nonpigmented, hence were overlooked, or because the roots of metaplastic lashes embedded in the tarsus were not completely excised. Some patients had epilated irritating lashes prior to surgery, even if instructed not to, thus contributing to postoperative lash regrowth.

The residual lashes <6 reduced over the follow-up period due to the adjuvant electrolysis. However, by the end of the 6 months, the actual rate of recurrence was 14.9% with lashes either in the original or in the new places and they were candidates for another surgical intervention mainly repeating ALR. A similar recurrence rate (11%) was reported in Koreen et al. study [14].

This recurrence rate is also comparable to BLTR procedure (7.4 to 63%) [10, 19] and PTLR (12% to 55%) [18]. In their retrospective study, Barr et al. found that the recurrence rates for TT treated by both BLTR and ALR showed no statistically significant difference. However, they found that in cases that had equal follow-up periods for both procedures, recurrence in ALR group is less [20]. Madarosis, persistent abnormal lid appearance, and recurrence are the common complications associated with ALR while overcorrection, granuloma formation, and notching ischemia of the lid margin and defective lid closure have also been more reported with tarsotomy in BLTR and PLTR [18, 21].

Although this work is a short term follow-up yet it should be noted that late recurrence is suggested to be due to an ongoing scarring process. West el al. [22] reported 7.6% recurrence rate in one year compared to 2.3% at 6 weeks in their study in Southern Ethiopia. The cumulative recurrence rate in Rajak et al. work increased from 32% in the first year to 41% in the 4th year [10]. This late recurrence is linked to inflammatory mediators IL-1B and genetic susceptibility bacterial reinfection while tumor necrosis factor TNF was linked to scarring [23].

Grey line splitting with anterior lamella repositioning has various modifications with tarsus fracture, wedge resection, putting a tarsus substitute, and use of everting sutures as well as use of mucous membrane to cover the bare tarsus [15]. Anterior lamellar reposition can also be carried out via skin crease incision in the upper lid. We believe that splitting starting from the lid margin provides more controlled placement of the incision behind the aberrant lash line before separating the two lamellae. Lash resection is also believed to augment the results [24].

Leaving the bare posterior lamella to granulate, though it gave the patient an odd appearance, provided time for the granulation tissue to cover the lash bearing area before the anterior lamella migrated back to its original place,. Persistence of the abnormal lid appearance was minimal and was due to delayed suture removal for these patients who were from border areas.

In conclusion, anterior lamellar recession without mucous membrane graft is a good option for treating trachomatous trichiasis especially in the absence of associated cicatricial entropion with good functional and cosmetic outcomes. The success rate is comparable to other BLTR and PTLR. Proper placement of the incision behind the aberrant lashes, visualizing their roots with excision of extra-lash bearing area, is believed to be of utmost importance in preventing recurrence. However, at least one adjunctive lash electrolysis or laser ablation session is usually required postoperatively.

Further studies are required to establish the factors affecting the outcome and to validate the value of excision of lash bearing area as well as comparing the outcome according to the surgical approach whether via skin crease or starting at the lid margin. Studies with longer follow-up periods are also recommended to evaluate the long lasting effect of this procedure.

## Figures and Tables

**Table 1:** Frequency and outcome of electrolysis per each follow-up.

Treatment	First session		Second session		Third session	
	Treated	Cured (%)	Treated	Cured (%)	Treated	Cured (%)
Three weeks	270	184 (68.1%)	NA	—	NA	—
Three months	80	55 (68.8%)	30	26 (86.7%)	NA	—
Six months	32	13 (40.6%)	18	12 (66.7%)	15	11 (73.3%)
